# Advances in stem cell therapy for diabetic foot

**DOI:** 10.3389/fgene.2024.1427205

**Published:** 2024-09-03

**Authors:** Yinfeng Xia, Ping Wu, Hong Chen, Zhiyong Chen

**Affiliations:** ^1^ Department of Burn and Plastic Surgery, Chongqing University Fuling Hospital, Chongqing University, Chongqing, China; ^2^ Department of Hepatobiliary Surgery, The Second Affiliated Hospital of Chongqing Medical University, Chongqing, China

**Keywords:** diabetic foot ulcers, stem cell therapy, angiogenesis, anti-inflammatory, wound healing

## Abstract

Diabetic Foot Ulcers (DFU) represent a grave complication often encountered in the advanced stages of diabetes mellitus. They frequently lead to recurrent hospitalizations and, in severe cases, can result in life-threatening conditions such as infections, gangrene, and even amputation Diabetic foot ulcers (DFU), as a serious complication in the late stage of diabetes mellitus, are prone to lead to repeated hospitalization, and in severe cases, infection, gangrene, and even amputation. Although there are many methods for treating diabetic foot, there is no clear and effective method to reduce the amputation rate of diabetic foot patients. In recent years, advancements in the understanding of stem cell therapy for the treatment of DFU have shed light on its potential as a novel therapeutic approach. In recent years, as the research on stem cell therapy for diabetic foot is gradually deepening, stem cells are expected to become a new therapeutic method for treating DFU in the future. Their therapeutic effects are through promoting angiogenesis, secreting paracrine factors, controlling inflammation, promoting collagen deposition, and regulating immunity, etc. Despite numerous studies confirming the efficacy of stem cell therapy in treating DFU, there is still a need for the establishment of standardized treatment protocols. Although numerous studies have shown that stem cell therapy for DFU is real and effective, there has not yet been a standardized treatment protocol. This article reviews studies related to stem cell therapy for DFU, looking at the mechanism of action, types of stem cells, and modes of administration.

## 1 Background

Diabetes Mellitus (DM) represents a significant public health concern and is among the most critical medical emergencies globally. The incidence of diabetes mellitus is gradually increasing and the incidence of the disease is becoming younger due to the rising standard of living in today’s society, the increasing pressure of life and work, and people’s neglect. Diabetes is a rapidly growing epidemic in most countries. DFU is its major complication and one of the leading causes of death in patients, with a global prevalence of approximately 1.8%–6.3% (Zhang et al., 2020; Zhang et al., 2017), and approximately 20% of patients are at risk of amputation (Ghotaslou et al., 2018; Lin et al., 2019). Of these, a substantial portion of lower extremity amputations can be traced back to diabetic foot ulcers, accounting for about 85% of cases (Armstrong et al., 2023). There are numerous causes of DFU, but the causes and mechanisms are not clearly defined. Most believe that the occurrence of DFU is mainly related to peripheral neuropathy, vasculopathy, and foot infection. Slow wound healing in DFU is thought to be related to its reduced angiogenesis, infiltration of inflammatory factors, and reduced collagen deposition (Mahmoudvand et al., 2023). Comorbid with DM, peripheral vascular disease is a significant source of morbidity and mortality among patients with DFU (Bassi et al., 2012). This is the reason why diabetes is considered a disease that can affect all sizes and types of blood vessels (Sasor and Ohlsson, 2014). Foot infections are also a common complication of the diabetic foot, which is related to the hyperglycemic environment of the trauma. Bacterial infections, including those caused by *Staphylococcus aureus* and *Pseudomonas aeruginosa*, are common in DFU and frequently necessitate antibiotic therapy and surgical debridement under negative pressure (Senneville et al., 2024; Banu et al., 2015). Currently, the main principles of Western medical treatment are glycemic control, infection control, removal of necrotic tissue, decompression and hyperbaric oxygen, and restoration of blood flow reconstruction (Reardon et al., 2020; Wenhui et al., 2021; Fan et al., 2020). However, for some distal arterial stenosis and occlusion, vascular intervention or vascular bypass bypass surgery cannot be performed (Houlind, 2020). What’s more, surgical treatment is not possible due to their condition. Therefore, there is an immediate requirement for the development of a novel therapeutic approach to the treatment of DFUs and to reduce their amputation rate.

In recent years, there has been a burgeoning interest in novel therapeutic modalities for the management of DFU, encompassing the local application of growth factors, the advent of novel biological dressings, and the burgeoning field of stem cell therapy. Previous studies have found that recombinant platelet-derived growth factor-enriched growth factors can be used to treat diabetic foot neuropathic ulcers by promoting cellular aggregation, angiogenesis, and cellular proliferation to accelerate the growth of granulation tissue in the repaired wound. Developed by tissue and bioengineering, many dressings have been invented that can carry drugs and growth factors. Some studies have shown that autologous platelet-rich gel can significantly shorten the course of the disease, increase the cure rate of DFU, and prevent infection (Li et al., 2015). Prussian blue nanoparticles (PBNP) is an iron-based material with good biocompatibility and a strong scavenging power of reactive oxygen species, and wound dressings containing PBNP can effectively promote the healing of DFU by reducing reactive oxygen production and promoting angiogenesis (Xu et al., 2022). Despite these promising developments, it is noteworthy that the International Diabetic Foot Working Group has expressed caution regarding the widespread use of agents that alter the wound microecology, such as growth factors and autologous platelet gels, due to a paucity of conclusive evidence from large-scale, randomized controlled trials (Rayman et al., 2020). The use of growth factors in isolation fails to account for the intricacies of the wound-healing process. Stem cells, on the other hand, are pivotal in this process, and stem cells can comprehensively regulate tissue regeneration by improving the wound microenvironment (Yu Q. et al., 2022). Many studies have shown that the use of stem cell transplantation is more effective compared to conventional therapy alone (Gao et al., 2019). Furthermore, the synergistic effects of combining traditional treatment protocols with stem cell therapy for DFU have also been documented, suggesting improved outcomes (Qin et al., 2016; Zhao et al., 2020). Although numerous studies have shown that stem cell therapy for DFU is safe and effective, the specific mechanisms and treatment modalities have not been fully clarified. Therefore, this review will describe the research progress of stem cell therapy for DFU in these two aspects.

## 2 Molecular mechanism of stem cell therapy for diabetic foot

### 2.1 Promoting angiogenesis

**FIGURE 1:**
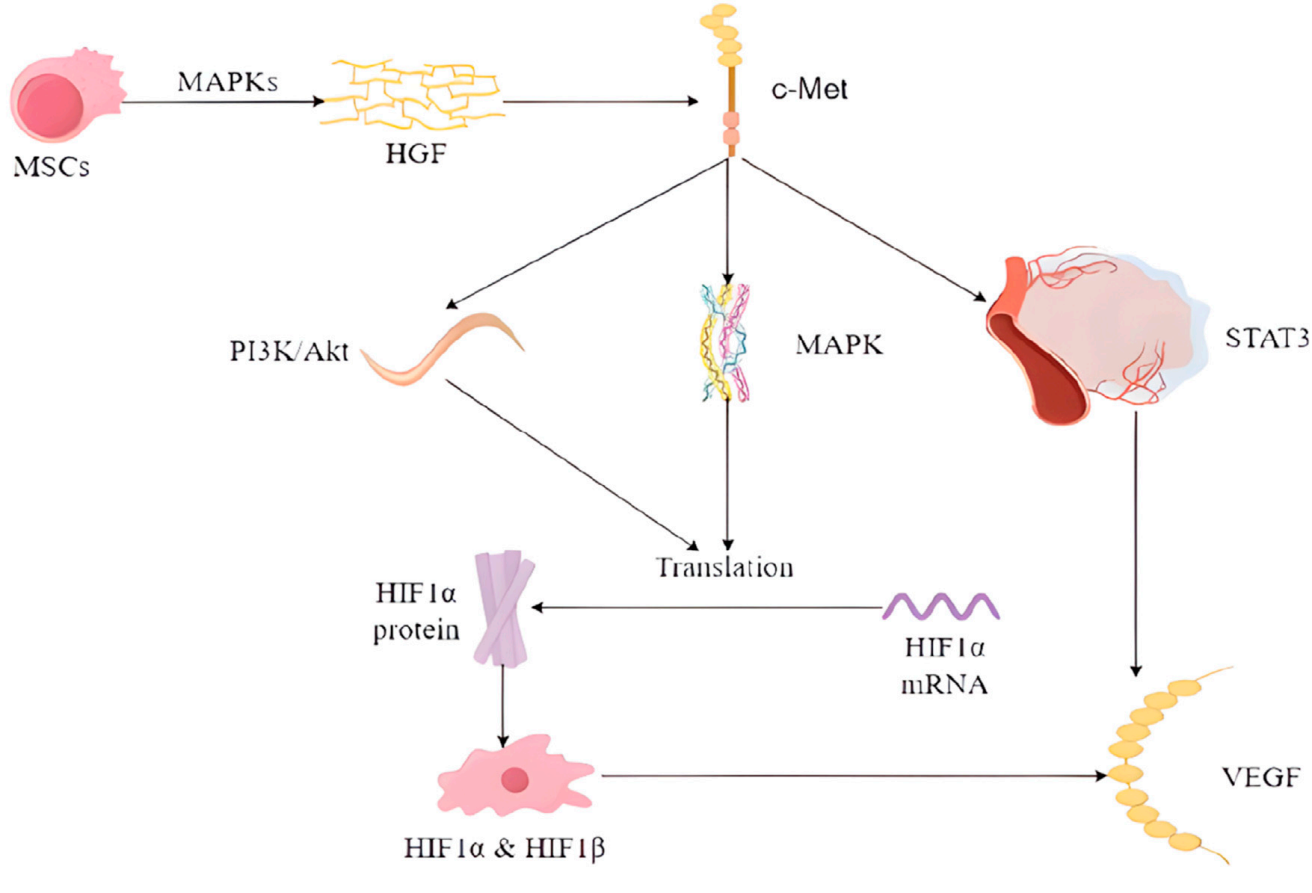
Mechanism of HGF and VEGF enhancement by MSCs.

Wound healing is a complex process, especially when the hyperglycemic environment affects wound angiogenesis. A synergistic interaction between multiple cells and cytokines is required for optimal results. Among these, vascular endothelial growth factor (VEGF) is of particular importance. Although the use of embryonic stem cells (ESCs) is limited for ethical reasons, previous animal studies have shown that they can increase the expression levels of fibronectin, VEGF, and epidermal growth factor (EGF) (Lee et al., 2011). With the progress of research on stem cells, it has been found that multipotent stem cells (MSCs) can differentiate into vascular endothelial cells, which is a key step in angiogenesis. They can activate specific signaling pathways, e.g., HGF binding to the c-Met receptor activates several signaling pathways including PI3K/Akt, MAPK, and STAT3, which are involved in the regulation of VEGF expression. (Matsumura et al., 2013). HMSCs have been observed to secrete VEGF and HGF through the signaling pathway that activates p38 mitogen-activated protein kinases (MAPKs) (Kopan and Ilagan, 2009) (Figure 1).

MSCs can systematically regulate injured and ischemic tissues, up-regulate cytokines and growth factors in them, and improve the microenvironment of the wounds, thus promoting the healing of diabetic foot wounds (Shi et al., 2020; Shi et al., 2016; Wan et al., 2013; Zhang et al., 2010). Cytokines and growth factors act through a variety of mechanisms, including modulation of immune cell activity and improvement of vascular permeability, and also act as signaling agents (Matthay et al., 2010). It has been demonstrated that VEGF stimulates the local upregulation of platelet-derived growth factor B (PDGF-B) and fibroblast growth factor 2 (FGF-2) in the context of trauma, promotes bone marrow-derived cell aggregation, and facilitates wound healing and tissue remodeling (Galiano et al., 2004; Ishida et al., 2019). Among them, endothelial progenitor cells (EPCs) and adult bone marrow-derived hematopoietic stem cells (BMHSCs), which can act as precursors of erythrocytes, platelets, and leukocytes, are introduced to the local microenvironment at the wound site, where they have been observed to facilitate the healing process and tissue regeneration (Yang J. et al., 2020). MALAT1 expression was found to be downregulated in patients with DFU (Jayasuriya et al., 2020) and microRNA-205-5p was found to be a direct regulator of VEGF protein translation, which can result in the inhibition of the translation of VEGF proteins, thereby impeding the healing of DFU (Rehak et al., 2022). While MALAT1 has a competitive relationship with microRNA-205-5p, MSCs will decrease the level of microRNA-205-5p and promote VEGF production by expressing elevated MALAT1 (Zhu et al., 2019). Furthermore, elevated MALAT1 levels have been observed to enhance the expression of collagen I and III in the skin of diabetic mice, resulting in increased collagen deposition at the wound site and accelerated wound healing (Liu et al., 2022), while collagen is secreted by fibroblasts (Vig et al., 2017). In addition, increasing local MALAT1 levels in wounds can greatly reduce inflammation and accelerate healing. For example, MALAT1 overexpression in MSCs induces M2 macrophage polarization and reduces the expression levels of pro-inflammatory cytokines IL-6 and TNF-α (Kuai et al., 2022). Therefore, MSCs can promote healing of DFU through the aforementioned pathways.

ADSCs have emerged as a promising therapeutic strategy for the treatment of diabetic foot ulcers, largely due to their ability to augment angiogenesis and the proliferation of endothelial progenitor cells through the expression of the transcription factor Nrf2 (Li et al., 2018). The transcription factor Nrf2 is dependent on heme oxygenase-1 (HO-1), which mainly acts to promote angiogenesis and increased expression of angiogenic factors (Loboda et al., 2016). The overexpression of HO-1 in BMMSCs has been demonstrated to promote BMMSCs proliferation and enhance VEGF secretion by BMMSCs through the Akt signaling pathway. This cascade of events culminates in a significant improvement in the repair of wound ulcers (Hou et al., 2013). MFG-E8 is essential for VEGF-induced AKT phosphorylation and is expressed both perivascularly and intravascularly. A study has confirmed that BMMSCs can overexpress MFG-E8 to enhance angiogenesis, further underscoring the potential of stem cell-based therapies in the treatment of chronic wounds like DFU.

In DFU, c-Jun expression was also decreased, and HUC-MSCs overexpressing c-Jun were found to accelerate angiogenesis and re-epithelialization by increasing PDGFA and HGF levels when injected locally subcutaneously into diabetic wounds (Yue et al., 2020; Tombulturk et al., 2019). Activator protein 1 (AP1) represents one of the principal downstream effectors of MAPKs (Kajanne et al., 2007). C-Jun, the primary component of AP-1, is a vital regulator of cellular proliferation (Davies et al., 2013). Studies have shown that MMP-2 and MMP-9, which are increased in diabetic chronic ulcers, can slow wound healing by degrading the skin’s extracellular matrix (ECM) (Wysocki et al., 1993; Krishnan et al., 2018). In contrast, it was demonstrated that AP-1 predominantly regulates MMP-2 and MMP-9 transcription in diabetic wounds (Yue et al., 2020). The overexpression of c-Jun in MSCs may facilitate wound repair by downregulating the expression of MMP-2 and MMP-9.

### 2.2 Control of inflammation and modulation of immunity to promote wound repair

Prolonged inflammation can create an environment with low oxygen levels and lead to the abnormal production of angiogenic signals (Ma et al., 2020). The anti-inflammatory and immunomodulatory effects of transplanted stem cells have been recognized as potential mechanisms for the restorative effects of stem cell therapy. The immune system is regulated by mild inflammation, which plays a crucial role in the elimination of pathogens, the acceleration of tissue repair, and the maintenance of homeostasis (Arabpour et al., 2021). However, persistent and severe inflammation is the primary cause of delayed or non-healing DFUs. It is not uncommon for immunosuppression and prolonged inflammation to coexist. Severe immune responses may result in the development of severe systemic inflammatory or allergic conditions, while reduced immune responses may lead to the occurrence of severe or recurrent infections. Therefore, there is a need to ensure that the pro- and anti-inflammatory aspects of the wound are in a balanced state. A study was conducted to investigate the efficacy of embryonic stem cell extracts in the treatment of diabetic foot ulcers. The results showed that the topical application of these extracts led to a decrease in CD45^+^ inflammatory cells and interferon-alpha (IFN-α), along with an increase in regulatory T cells (Tregs), proliferating Ki-67+ cells, and the endothelial cell marker CD31 (Loretelli et al., 2020). Suggesting that ESCs can promote wound healing by modulating the immune response. Previous studies have shown that MSCs can reduce local pro-inflammatory responses and inhibit CD45 expression, demonstrating the anti-inflammatory effects of MSCs in repairing DFU (Kuo et al., 2011; Yang H. Y. et al., 2020).

Reduced levels of CCL2 are observed in DFU, and its enhanced expression can facilitate wound healing through the normalization of neovascularization and the accumulation of collagen (Ishida et al., 2019). The expression of CCL2 was found to be significantly elevated following the local administration of MSCs (Yang J. et al., 2020). CCL2 receptors are expressed on macrophages, which secrete VEGF and TGF-β, and the levels of macrophages are reduced in DFUs. In contrast, some studies have shown that MSCs can increase VEGF and TGF-β levels at diabetic wound sites. Therefore, local application of stem cells may promote macrophage aggregation and growth of VEGF and TGF-β by increasing CCL2. The transcription factor Nrf2 directly regulates the secretion of CCL2 by epidermal keratinocytes. In turn, CCL2 can regulate the production of EGF in macrophages at the site of injury, which in turn stimulates the proliferation of keratinocytes (Villarreal-Ponce et al., 2020). Therefore, it is postulated that MSCs may enhance CCL2 secretion by facilitating functional recovery of keratinocytes, thereby reversing the reduction in macrophage infiltration and ultimately promoting ulcer repair. Moreover, hyperglycemia has been shown to reduce the number of endothelial progenitor cells (EPCs), compromising their function and their ability to aggregate (Tepper et al., 2002). The elevated expression of CCL2 in diabetic mice further enhances the accumulation of EPCs at wound sites, thereby promoting neointimal formation (Ishida et al., 2019).

Chronic non-healing wounds in diabetes are distinguished by an elevation in pro-inflammatory cytokines, which include IL-1, IFN-γ, TNF-α, and IL-6. These cytokines are predominantly produced by activated macrophages and play a critical role in the regulation of immune cells (Zubair and Ahmad, 2019). Local application of MSCs may attenuate the inflammatory response by secreting IL-10. It was found that treatment with placenta-derived mesenchymal stem cells (PMSCs) combined with IL-10 antibody significantly slowed down the healing of DFUs (Wang et al., 2016). A strong inhibition of lipopolysaccharide (LPS)-induced NF-kB activation in dermal fibroblasts by PMSCs was observed (Varela et al., 2019). While NF-kB plays a crucial role in regulating the production of pro-inflammatory cytokines such as IL1, TNF-α, and IL-6, mesenchymal stem cells (MSCs) facilitate cutaneous wound healing by reducing the release of pro-inflammatory cytokines and promoting the production of anti-inflammatory cytokines in the wound area (Shen et al., 2021).

There are many types of macrophages, and different phenotypes have different roles (Xia et al., 2021). Macrophages can be classified into two main phenotypes: The two main categories are M1 and M2. M1 macrophages are activated by proinflammatory cytokines. Conversely, anti-inflammatory cytokines can induce the transformation of macrophages from the M1 to the M2 phenotype (Krzyszczyk et al., 2020). Studies have shown that M2 macrophages promote angiogenesis, reduce nerve damage, and inhibit inflammation, and topical application of SCs to wounds has been shown to induce a macrophage shift toward an anti-inflammatory phenotype (M2) (Boodhoo et al., 2022).

## 3 Cellular mechanisms

### 3.1 Differentiated cells and pro-collagen deposition

Wound healing is a complex process that includes periods of homeostasis, inflammation, proliferation, and remodeling (Mahmoudvand et al., 2023; Bray et al., 2021). In the first two stages, MSCs promote coagulation and control inflammation. In the proliferative phase, MSCs also play a crucial role. During this phase, epithelial cells undergo proliferation and repair and synthesize collagen and other ECM proteins. (Andalib et al., 2023) MSCs are precursor cells with the ability to self-regulate and proliferate (Afkhami et al., 2023). HMSCs can actively differentiate into epithelial and endothelial cells, and HMSCs share common properties with ADSCs that enable them to develop into endothelial cells and neovascularization (Caplan and Dennis, 2006). The specific pathways by which they are transformed into endothelial cells have not been fully characterized. It has been shown that the MAPK/ERK signaling pathway promotes VEGF-induced differentiation of BM-MSC to endothelial cells, whereas the PI3K signaling pathway regulates the differentiation of ADSC to endothelial cells (Yu X. T. et al., 2022).

**FIGURE 2:**
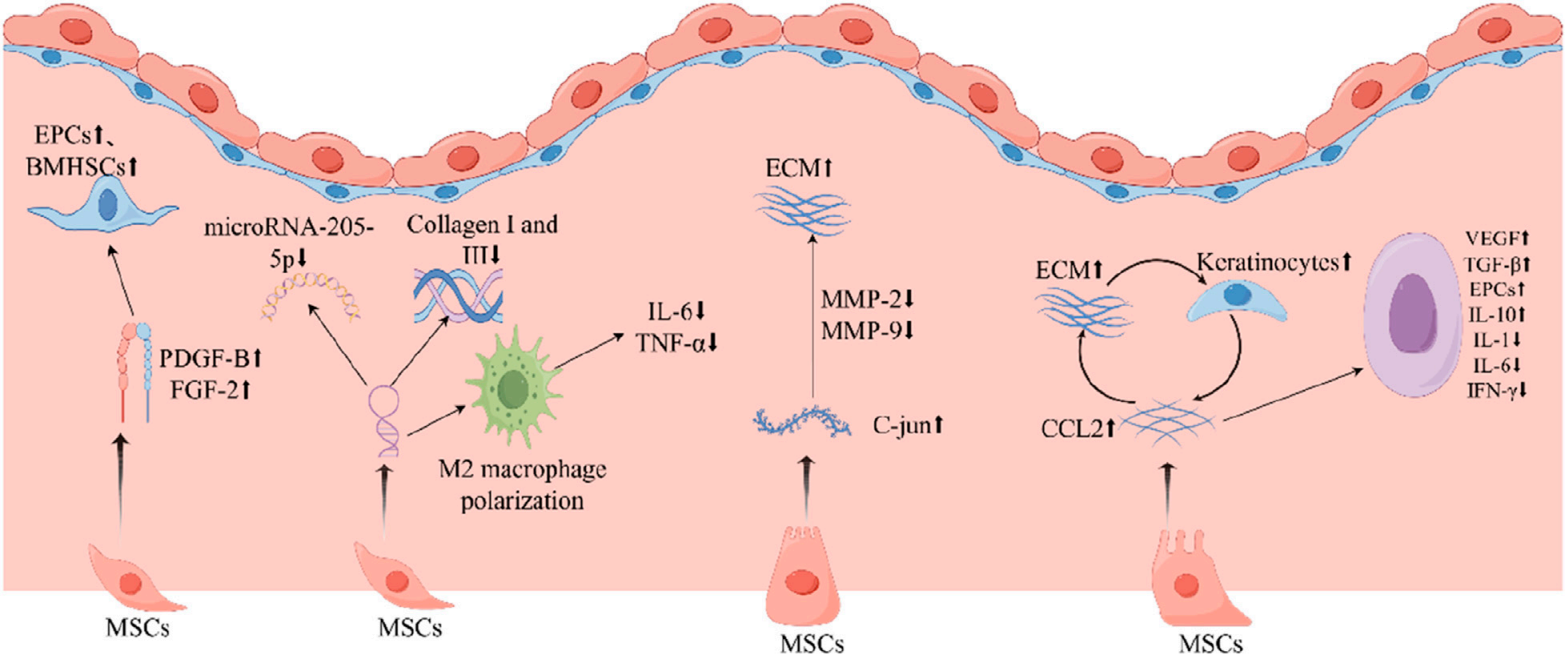
MSCs play an important role in repairing diabetic foot ulcers. MSCs, mesenchymal stem cells; IL-1, interleukin-1; IL-6, interleukin-6; IL-10, interleukin-10; TNF-α, tumor necrosis factor-α; TGF -β, transforming growth factor-β; EGF, epidermal growth factor; EPCs, endothelial progenitor cells; ECM, extracellular matrix; FGF-2, fibroblast growth factor 2; PDGF-B, platelet-derived growth factor B; IFN-γ, interferon-γ; VEGF, vascular endothelial growth factor.

Collagen production and distribution are mainly found in the dermis of the skin, released by fibroblasts, and are closely related to the healing of skin injuries. MSCs may increase collagen production and promote collagen formation in DFU (Lin et al., 2022). ADSCs in diabetic foot wounds are directly transformed into fibroblasts and significantly promote the expression of type I collagen and type III collagen (An et al., 2020). It has been demonstrated that fibroblasts derived from diabetic foot tissue exhibit a reduced proliferative capacity and the production of fewer growth factors (Zeidi et al., 2020). HMSCs can significantly increase ECM production in diabetic fibroblasts. Consequently, MSCs may facilitate granulation tissue growth by stimulating fibroblast proliferation and functional recovery. This, in turn, stimulates the release of additional ECM and growth factors from fibroblasts, thereby promoting tissue repair (Shi et al., 2020) (Figure 2) (Table 1).

**TABLE 1:** Molecular and cellular mechanism of stem cell therapy for diabetic foot.

		
Molecular mechanism	Promoting angiogenesis	Activates multiple signaling pathways including PI3K/Akt, MAPK, and STAT3; increase VEGF, HGF, EGF levels
		Overexpression of MALAT1, HO-1, MFG-E8, C-jun
	Control of inflammation and modulation of immunity	Reduction of CD45^+^, IFN-α. Increase in regulatory T cells (Tregs), proliferating Ki-67^+^ cells, and the endothelial cell marker CD31
		Increase the expression level of CCL2
		Promotes the release of anti-inflammatory cytokines
		Reduced levels of IL-1, IFN-γ, TNF-α, IL-6
		Promoting M1 to M2 phenotype transition
Cellular mechanism		Differentiation into epithelial and endothelial cells
		Increased ECM deposition
		Stimulates fibroblast proliferation and functional recovery

## 4 Classification of stem cells

### 4.1 Mesenchymal stem cells (MSCs)

MSCs are widely present in widely distributed connective tissues and organ mesenchyme and can be extracted from bone marrow, fat, placenta, umbilical cord, and amniotic fluid. It is the most commonly used stem cell type in research due to its huge content in the human body, easy accessibility, and economy. Among them, bone marrow mesenchymal stem cells (BMMSCs) and adipose-derived mesenchymal stem cells (ADSC) are the longest-used stem cell sources. It has been found that MSCs can populate the dermis of the skin and undergo phenotypic changes or senescence in chronic wounds (Blumberg et al., 2012). Given the high plasticity of bone marrow cells, it is postulated that they have the potential to generate new skin cells (Badiavas and Falanga, 2003). A substantial body of evidence indicates that the administration of BMMSCs can effectively enhance wound healing and facilitate the reconstruction of damaged skin, and its treatment of chronic non-healing wounds is a safe and effective treatment when the larger the wound, the more cells are needed (Dash et al., 2009; Falanga et al., 2007; Jain et al., 2011). When DFU is combined with lower limb ischemia, peripheral blood-derived MSCs can effectively promote wound healing and reduce the amputation rate. The treatment was administered intramuscularly into the ischemic lower limbs and feet of patients with DFU and lower limb ischemic disease. The results demonstrated that the intervention was capable of preventing foot and lower limb amputation and improving quality of life. Furthermore, the method may be considered safe and effective for the treatment of lower limb ischemic disease (Yang et al., 2005). In contrast, adipose-derived mesenchymal stem cells (AMSCs) are considered an ideal cell-based treatment for chronic diseases due to their superficial location, easy availability, abundant sources, approximately threefold increase in immunosuppressive activity compared to BMSCs, and due to the greater cell division capacity of AMSCs (Hassanshahi et al., 2019; Kokai et al., 2014; Qi et al., 2019). The ethical aspects are better passed since AMSCs are obtained from autologous adult fat (Palumbo et al., 2018). Furthermore, several studies have demonstrated that AMSCs are capable of facilitating wound healing by stimulating the formation of epithelial and granulation tissue, through their anti-inflammatory and anti-apoptotic effects, as well as pro-angiogenesis (Gadelkarim et al., 2018; Álvaro-Afonso et al., 2020). However, the utilization of autologous AMSCs in cell-based therapy for diabetic patients is constrained by the effects of diabetes. Consequently, interventions are required to enhance cellular function before application (Rennert et al., 2014). For example, adding platelet-rich plasma to AMSCs, embedding AMSCs in autologous platelet-rich fibrin (PRF), etc (Tobita et al., 2015; Sharma et al., 2022). Human umbilical cord mesenchymal stem cells (HUCMSCs) are a type of progenitor cells with high differentiation potential, which can promote the formation of new blood vessels and improve tissue regeneration (Yan et al., 2022). Moreover, its derived exosomes are stable and immunogenic, capable of transporting multi-functional proteins and growth factors with different roles, and not only that, it can regulate the proliferation and differentiation of BMMSCs (Zhang et al., 2021; Wen et al., 2020). Although bone marrow is currently the main source of MSCs, numerous studies have demonstrated that placental-derived stem cells (PDMSCs) may be a superior alternative. It is readily available, can isolate numerous cells, and is less immunogenic with less rejection (Chang et al., 2006; Beeravolu et al., 2017). In addition to this, PDMSCs can differentiate into a multitude of cell types (Wang et al., 2016; Beeravolu et al., 2017; Fukuchi et al., 2004).

### 4.2 Mononuclear cells (MNC)

MNCs are generally categorized into two main sources: bone marrow and peripheral blood. Both types possess the capacity for self-renewal, differentiation, and proliferation. They can be further distinguished into specific cell types, such as smooth muscle cells and vascular endothelial cells, under certain conditions, and can also secrete cytokines to promote wound healing. A clinical study demonstrated the safety and efficacy of autologous peripheral blood MNC in the treatment of DFU. The trial revealed that the application of autologous peripheral blood MSCs significantly decreased the amputation rate in patients with DFU (Scatena et al., 2021). Nevertheless, additional randomized studies have been conducted to assess the efficacy of intramuscular injections of BMMNC and BMMSCs in the treatment of chronic wounds in patients with diabetic critical limb ischemia and foot ulcers. The results demonstrated that while both methods reduced pain and improved ankle-brachial index (ABI) and transcutaneous partial pressure of oxygen (tcPO2), perfusion BMMNCs were less effective than BMMSCs in promoting wound healing and improving perfusion (Lu et al., 2011). This finding could also explain why there is currently a greater focus on MSC research.

### 4.3 Embryonic stem cells (ESCs) and induced pluripotent stem cells (iPSCs)

Embryonic stem cells (ESCs) are pluripotent; they are isolated from the inner cell population of blastocysts in early development or from *in vitro* fertilized embryos (Uzun et al., 2021; Bogliotti et al., 2018; Salari et al., 2023). With self-renewal ability, they can differentiate into any cell lineage. It has been demonstrated that the topical administration of undifferentiated ESCs expedites wound healing in diabetic mice, promotes secretion of growth factors, re-epithelialization, and accelerates wound healing, and its extracts have anti-inflammatory and immunomodulatory properties (Lee et al., 2011; Loretelli et al., 2020). However, due to its ethical implications and its excessive proliferation and differentiation ability, it may lead to immune rejection or induce tumor formation (Jiang et al., 2012).

Similar to ESCs, iPSCs have pluripotency and self-renewal ability, as well as the potential to differentiate into various cell types of organism. They can theoretically be obtained from various adult tissues, including skin, and are therefore less ethically controversial than ESCs, and iPSCs are more advantageous than other stem cells in tissue regeneration and chronic wound healing (Lopes et al., 2018). iPSC-derived terminally differentiated cells can compensate for the insufficient levels of cytokines in patients with diabetic foot by secreting a variety of growth factors and cytokines which in turn promotes wound healing (Clayton et al., 2018; Wang, 2021). However, the research of iPSC in the fields of three-dimensional printing of organs, wound healing, and angiogenesis is still in the preclinical stage, and its safety needs to be further evaluated if it may be used in clinic (Table 2).

**TABLE 2:** The role of stem cells in the treatment of diabetic foot wounds.

References	Cell type	Mode of administration	Outcome
Badiavas and Falanga (2003)	BMMSCs	Topical application and subdermal injection	Wound reduction, increased dermal blood flow and thickness
Dash et al. (2009)	BMMSCs	Topical application and subdermal injection	Reduced ulcer size and pain
Jain et al. (2011)	BMMSCs	Topical application and subdermal injection	Increased rate of ulcer healing
Rennert et al. (2014)	ASCs	Subdermal injection	Decreased angiogenesis in diabetic mice
Yan et al. (2022)	HUCMSCs	Subdermal injection	Inhibits inflammation, promotes angiogenesis and wound healing
Zhang et al. (2021)	UCMSCs	Intravenous injection	Regulation of the TGF-β signaling pathway. Reduce wound size
Scatena et al. (2021)	PBMNCs	Subcutaneous injection and intramuscular injection	Significantly reducing the amputation rate and improving survival and wound healing.
Lu et al. (2011)	BMMSCs BMMNCs	Intramuscular injection	The ulcer healing rate of the BMMSC group was significantly higher than that of BMMNCs
Uzun et al. (2021)	Allogeneic ASCs	Subdermal injection	Reduced wound size and safety
Clayton et al. (2018)	iPSCs	Subdermal injection	Promote angiogenesis, fibroblast infiltration and collagen deposition, accelerate wound closure

## 5 Route of administration

Stem cell therapy can be broadly categorized into two primary methods: local and systemic administration. A comprehensive review of the literature indicates that both pathways promote DFU healing. Typically, local injections are the most common method of cell delivery. In clinical studies, intramuscular injections are the most commonly used, while in preclinical studies, intradermal and subcutaneous injections are the most prevalent (Lopes et al., 2018). Compared with intravenous administration, local injection reduces the link between stem cells undergoing chemotaxis and homing to the target organ, effectively increasing the concentration of stem cells in the target organ. This method can also expedite the healing process and improve graft survival by stimulating the secretion of extracellular matrix and the regeneration of tissue (Held et al., 2016; Jung et al., 2010). In the systemic route, stem cells can be administered arterially or intravenously. It has been shown that this approach has immunomodulatory effects and can also regulate blood glucose (Yan et al., 2021). However, intravascular administration increases the risk of microthrombosis and may be related to the dose administered. In contrast to local injections and intravenous treatments, topical wound application of drugs has a better safety profile and is more direct and convenient. Topical application of stem cell preparations to the wound in combination with emerging vectors that enhance stem cell activity and better control cell spacing is a new direction in the treatment of DFU (Lopes et al., 2018; Amer et al., 2018). A plethora of studies have substantiated that topical administration is not only safer but also more effective than systemic administration.

## 6 Discussion and conclusion

To summarize the results of the study indicate that stem cells have a promising application in the treatment of DFU. They mainly play a role by promoting angiogenesis, secreting growth factors, stimulating vascular differentiation, inhibiting inflammation, promoting collagen deposition, and immunomodulation. It is shown that stem cells are effective in diabetic wound healing and reducing the amputation rate. However, most of the current studies are preclinical, and clinical randomized controlled studies with large samples are needed in the future to verify their efficacy. In particular, the comparative efficacy of its different stem cells, the route of administration, and the dosage of administration should be explored to find a standardized treatment regimen.

In addition, while the efficacy of stem cells is exciting, there are many risks associated with them. For example, firstly, immune rejection, although rejection can be reduced by immunosuppressive treatments, there is a corresponding increase in the risk of infection; secondly, the risk of tumorigenicity, the uncontrolled proliferation regulation and immunomodulation of stem cells facilitates tumor formation, and their secretion of a variety of growth factors to promote neovascularization and immune modulation promotes tumorigenesis. In addition, there is a need to develop appropriate diagnostic and treatment standards as well as laws and regulations on stem cell production to ensure clinical feasibility.

Diabetic foot ulcers can be treated in a variety of ways, and a combination of therapies is more effective in treating the diabetic foot. For example, in order to improve the survival rate of stem cell transplantation, it is possible to carry out hypoxic pretreatment, and the use of stem cell scaffolding complexes, combined PRP and biomaterials is more conducive to the healing of diabetic foot ulcer wounds. In conclusion, stem cell therapy for diabetic foot has a promising future, but more efficient preparation methods are needed, and further research is needed to determine the optimal type of stem cells needed for treatment, their safety, appropriate dosage, and the most effective route of administration.
