# Corrigendum: Advances in stem cell therapy for diabetic foot

**DOI:** 10.3389/fgene.2024.1520519

**Published:** 2024-12-12

**Authors:** Yinfeng Xia, Ping Wu, Hong Chen, Zhiyong Chen

**Affiliations:** ^1^ Department of Burn and Plastic Surgery, Chongqing University Fuling Hospital, Chongqing University, Chongqing, China; ^2^ Department of Hepatobiliary Surgery, The Second Affiliated Hospital of Chongqing Medical University, Chongqing, China

**Keywords:** diabetic foot ulcers, stem cell therapy, angiogenesis, anti-inflammatory, wound healing

In the published article, there was an error in **Affiliation 1**. Instead of “^1^Department of Burn and Plastic Surgery, Fuling Central Hospital, Chongqing University, Chongqing, China” it should be “^1^Department of Burn and Plastic Surgery, Chongqing University Fuling Hospital, Chongqing University, Chongqing, China.”

The authors apologize for this error and state that this does not change the scientific conclusions of the article in any way. The original article has been updated.

